# Methods to extract and analyze fluid from human pluripotent stem cell-derived choroid plexus organoids

**DOI:** 10.3389/fnmol.2023.1243499

**Published:** 2024-01-29

**Authors:** Leon H. Chew, Eloi Mercier, Jason C. Rogalski, Sara Pippard, Erin Knock

**Affiliations:** ^1^STEMCELL Technologies, Vancouver, BC, Canada; ^2^Department of Biochemistry and Molecular Biology, University of British Columbia, Vancouver, BC, Canada

**Keywords:** choroid plexus, organoid, organoid cell culture, cerebrospinal fluid, pluripotent stem cell (PSC), proteomics, mass spectrometry

## Abstract

The choroid plexus (ChP) is a highly vascularized tissue lining the ventricular space of the brain. The ChP generates cerebrospinal fluid (CSF) and forms a protective barrier in the central nervous system (CNS). Recently, a three-dimensional human pluripotent stem cell (hPSC)-derived ChP organoid model has been developed. This model generates cystic structures that are filled with a fluid resembling CSF and are surrounded by an epithelial layer expressing ependymal choroid plexus-specific markers. Here we describe a method to generate these choroid plexus organoids using a commercial kit and methods to extract the CSF-like fluid for use in downstream analysis.

## 1 Introduction

The choroid plexus (ChP) is a vital component of the central nervous system located within the lateral, third, and fourth ventricles of the brain (Saunders et al., 2023). The primary function of the ChP is the production of cerebrospinal fluid (CSF), which fills the ventricles and surrounds the brain and spinal cord. CSF acts to cushion the brain, remove waste products, regulate brain temperature, and provides a medium for the exchange of nutrients and chemicals between the blood and brain tissue (Saunders et al., 2023). ChP tissues consist of a layer of specialized cells known as ChP ependymal cells (Vaughan and Peters, 1974). These cells are in close association with blood vessels, and together, form the blood-CSF barrier. This barrier allows nutrient exchange to selectively occur between the bloodstream and CSF. As a selective barrier, the ChP prevents the entry of potentially harmful substances and maintains homeostasis of the brain microenvironment. The ChP and blood-CSF barrier have recently gained attention for their involvement in various neurological processes, including the regulation of neuroimmune function (Zhu et al., 2018), brain development (Huang et al., 2010; Johansson et al., 2013), implicated in neurological disorders (Van Hoecke et al., 2021), as well as playing a role in memory and aging (Iram et al., 2022).

Traditionally, the ChP has been studied in animal models or immortalized cell lines (Monnot and Zheng, 2013; Delery and MacLean, 2019; Dani et al., 2021). These, however, are limited in their capacity to recapitulate all aspects of human-specific ChP biology or maintain the correct apico-basal polarity required to form a barrier in order to study the secretory functions of the ChP (Monnot and Zheng, 2013; Delery and MacLean, 2019; Dani et al., 2021). Previous methods to generate ChP tissue from human pluripotent stem cells (hPSCs) have shown success in generating cell types which express the appropriate ChP ependymal cell markers, such as TTR, APQ1, and CLIC6 (Watanabe et al., 2012). However, these models were unable to be used as a robust tool to study CSF secretion and barrier function of the ChP.

Recently, a method was developed to generate hPSC-derived ChP organoids (Pellegrini et al., 2020). The organoids formed well-defined cystic compartments filled with human CSF-like fluid and were also used to predict the permeability of neuroactive drugs across this ChP barrier. Proteomic analysis of the fluid extracted from ChP organoids revealed remarkable similarity to *in vivo* derived CSF samples. Interestingly, clinically relevant biomarkers such as APOE, insulin-like growth factor binding protein 7 (IGFBP7), and serpin family F member 1 (SERPINF1) were found in high abundance in fluid derived from ChP organoids (Pellegrini et al., 2020). Here, we will describe the generation of ChP organoids using a commercially available kit, discuss two methods for fluid extraction, and the results of proteomic analysis of the fluid.

## 2 Materials

### 2.1 Materials for choroid plexus organoid generation

• Dulbecco’s phosphate-buffered saline (D-PBS) (without Ca++ and Mg++) (STEMCELL Technologies, Canada, Catalog #37350)
• Gentle Cell Dissociation Reagent (STEMCELL Technologies, Canada, Catalog #100-0485)
• Y-27632 (STEMCELL Technologies, Canada, Catalog #72302)
• Trypan Blue (STEMCELL Technologies, Canada, Catalog #07050)
• Hausser Scientific™ Bright-Line Hemocytometer (STEM CELL Technologies, Canada, Catalog #100-1181)
• Corning^®^ Matrigel^®^ hESC-Qualified Matrix (Corning, USA, Catalog #354277)
• Costar^®^ 24-Well Flat-Bottom Plate, Tissue Culture-Treated (Corning, USA, Catalog #38017)
• Costar^®^ 24-well Ultra-Low Attachment Multiple Well Plates (Corning, USA, Catalog #3473)
• Corning^®^ 96-well Clear Round Bottom Ultra-Low Attachment Microplate (Corning, USA, Catalog #7007)
• 6-Well Ultra-Low Adherent Plate for Suspension Culture (STEMCELL Technologies, Canada, Catalog #100-0083)
• Axygen™ 200 μL Wide Bore Universal Pipetter Tips (Fisher Scientific, USA, Catalog #14-222-730)
• 100 mm Dish, Non-Treated (STEMCELL Technologies, Canada, Catalog #38045)
• Conical tubes, 50 mL (STEMCELL Technologies, Canada, Catalog #38010)
• Serological Pipettes, 5 mL or 10 mL (STEMCELL Technologies, Canada, Catalog #38003 or Catalog #38004)
• Organoid Embedding Sheet (STEMCELL Technologies, Canada, Catalog #08579 or Parafilm^®^)
• Celltron Orbital shaker (INFORS HT, Switzerland, Catalog #69455)
• Sterile forceps

### 2.2 Materials for CSF-like fluid extraction

• Dulbecco’s phosphate-buffered saline (without Ca++ and Mg++) (STEMCELL Technologies, Canada, Catalog #37350)
• Syringe (1 mL or 3 mL) (Covidien, Ireland, Catalog #8881501400 or #8881513934)
• Needle (28G or smaller), e.g., (BD Eclipse™, USA, Catalog #305757)
• Amicon^®^ Ultra-0.5 Centrifugal Filter Unit (Amicon, USA, Catalog #UFC5100)
• 1.5 mL microcentrifuge collection tubes
• Sterile spatula

### 2.3 Materials for western blot ofCSF-like fluid

• 4–15% Mini-PROTEAN^®^ TGX™ Precast Gel, 10-well, 50 μl (Bio-Rad, USA, Catalog #456-1084)
• 10X Tris/Glycine/SDS Running Buffer (Bio-Rad, USA, Catalog #161-0732)
• 4X Laemmli Sample Buffer (Bio-Rad, USA, Catalog #161-0747)
• 2-Mercaptoethanol (Sigma-Aldrich, USA, Catalog# 63689)
• Precision Plus protein All blue Prestained Protein Standards (Bio-Rad, USA, Catalog #161-0373)
• Imperial Protein Stain (Thermo Scientific, USA, Catalog #24615)
• Western Blot Transfer Buffer:
  ○ 48 mM Tris
  ○ 39 mM glycine
  ○ 0.0375% sodium dodecyl sulfate (SDS)
  ○ 20% methanol
• Methanol (ACS reagent, ≥ 99.8%) (Sigma-Aldrich, USA, Catalog #179337-4L)
• ImmunoBlot PVDF Membrane (0.2 μm) (Bio-Rad, USA, Catalog #162-0177)
• Extra Thick Blot Paper–MiniBlot Size (7 × 8.4 cm) (Bio-Rad, USA, Catalog #1703967)
  Western Blot Blocking Buffer:
  ○ 5% bovine serum albumin
  ○ 0.2% Tween 20 in PBS
• Wash Buffer:
  ○ 1X PBS with 0.1% Tween 20
• Clarity Western ECL Substrate (Bio-Rad, USA, Catalog #1705061)
• Mini-PROTEAN^®^ Tetra Vertical Electrophoresis Cell for Mini Precast Gels, 4-gel (Bio-Rad, USA, Catalog #1658004)
• PowerPac™ 300 Power Supply (Bio-Rad, USA, Catalog #1645052)
• *Trans-*Blot^®^ SD Semi-Dry Electrophoretic Transfer Cell (Bio-Rad, USA, Catalog #1703940)
• Gel Doc™ XR + System (Bio-Rad, USA, Catalog #1708170)

### 2.4 Materials for proteomic analysis by mass spectrometry

• Ammonium Bicarbonate–(Fisher, USA, Catalog #BP2413-500)
• Dithiothreitol (DTT) (Fisher, USA, Catalog #BP172-25)
• Chloroacetamide (CAA) (Acros Organics Catalog #1484150 000)
• Trypsin (NEB, USA, Catalog #P8101S)
• TFA l LC/MS grade (Sigma, USA, Catalog #80457-10m)
• C18 Desalting columns (Polygoprep Catalog #711720.1000) C18 powder, and (VWR, Catalog #CA55004-098)–3M Empore, 3M Empore C18 Extraction Disks, p/n: 2215
• Methanol (Fisher Catalog #A452-4)
• Acetonitrile (Fisher Catalog #A998-4)
• Formic Acid (Fisher Catalog #270480250)
• nanoElute^®^ Nano-Flow UHPLC system (Bruker Daltonics, USA)
• Gen2 25 cm Aurora Series analytical column with Captive Spray Insert (CSI; Ion Opticks #AUR2-25075C18A-CSI).

## 3 Methods

### 3.1 Method to generate choroid plexus organoids

**FIGURE 1:**
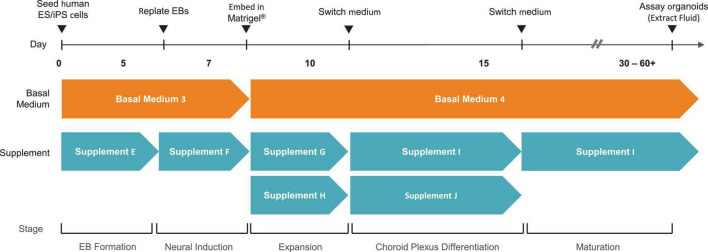
Overview of Method to Generate Choroid Plexus Organoids using the STEMdiff™ Choroid Plexus Organoid Kit.

Choroid plexus organoids are generated using a commercially available kit from STEMCELL Technologies (Figure 1) which is based on a technology licensed from the University of Cambridge (Pellegrini et al., 2020). For the purpose of drug screening applications, reproducibility is critical between different batches of organoids generated. Thus, we recommend the use of reagents developed using pre-screen raw materials and rigorously tested for performance, to allow for reproducible ChP organoid generation across multiple cell lines.

#### 3.1.1 Preparation of media

Use sterile technique to prepare STEMdiff™ choroid plexus organoid media. Prepare each medium as indicated in Table 1.

1. Thaw Supplement(s) at room temperature (15–25°C). Mix thoroughly.
  NOTE: If not used immediately, aliquot Supplement(s) and store at −20°C. Do not exceed the shelf life of the Supplement(s). After thawing aliquots, use immediately. Do not re-freeze.
2. Add Supplement(s) to Basal Medium as indicated in Table 1. Mix thoroughly. Warm complete medium to room temperature before use.
  NOTE: If not used immediately, store complete medium as indicated in Table 1.

**TABLE 1:** Preparation of STEMdiff™ Choroid Plexus Organoid Media.

Medium	Component	Volume	In-use storage and stability
Formation Medium (50 mL)	STEMdiff™ Neural Organoid Basal Medium 3	40 mL	Store at 2–8°C for up to 2 weeks.
	STEMdiff Neural Organoid Supplement E	10 mL	
Induction Medium (50 mL)	STEMdiff Neural Organoid Basal Medium 3	49.5 mL	Store at 2–8°C for up to 2 weeks.
	STEMdiff™ Neural Organoid Supplement F	0.5 mL	
Expansion Medium (25 mL)	STEMdiff™ Neural Organoid Basal Medium 4	24.25 mL	Store at 2–8°C for up to 2 weeks.
	STEMdiff™ Neural Organoid Supplement G	0.25 mL	
	STEMdiff™ Neural Organoid Supplement H	0.5 mL	
Choroid Plexus Differentiation Medium (50 mL)	STEMdiff™ Neural Organoid Basal Medium 4	48.5 mL	Store at 2–8°C for up to 2 weeks.
	STEMdiff™ Neural Organoid Supplement I	1 mL	
	STEMdiff™ Neural Organoid Supplement J	0.5 mL	
Maturation Medium (100 mL)*	STEMdiff™ Neural Organoid Basal Medium 4	98 mL	Store at 2–8°C for up to 2 weeks.
	STEMdiff™ Neural Organoid Supplement I	2 mL	

*Add 1% Corning^®^ Matrigel^®^ (v/v) to Maturation Medium. Media containing Corning^®^ Matrigel^®^ must be kept at 4°C at all times to avoid the formation of precipitate.

#### 3.1.2 hPSC quality

It is important to initiate ChP organoid formation using high-quality, undifferentiated hPSCs. hPSC cultures are ready to passage when the majority of colonies appear large, compact, and display dense, multi-layered centers with distinct borders. Passage hPSC cultures once they reach 70–80% confluency and exhibit < 10% spontaneous differentiation. hPSCs can be maintained in a variety of maintenance media, including mTeSR1™, mTeSR™ Plus, or TeSR™-E8™. We recommend passaging hPSCs as clumps (50–200 μm in diameter) using either non-enzymatic reagents, such as Gentle Cell Dissociation Reagent (STEMCELL Technologies, Canada, Catalog #100-0485), ReLeSR (STEMCELL Technologies, Canada, Catalog #05872), or enzymatic reagents such as Dispase (1 U/mL; STEMCELL Technologies, Canada, Catalog #07923). Check the karyotype routinely to ensure that hPSCs retain a normal karyotype over long-term culture. hPSC Genetic Analysis Kit (STEMCELL Technologies, Canada, Catalog #07550) can be used to frequently screen hPSCs for common karyotypic abnormalities that arise during routine cell culture. Undifferentiated hPSCs can also be assayed by immunocytochemical analysis of markers of the undifferentiated state, such as OCT4 and TRA-1-60. The individual cells should be tightly packed, exhibit a high nuclear-to-cytoplasm ratio, and have prominent nucleoli.

#### 3.1.3 Formation (Day 0–5)

This protocol is for the formation of organoids from a hPSC culture in a single well of a 6-well plate. For other cultureware, adjust volumes accordingly. Warm cultureware, media, and reagents to room temperature (15–25°C) before use.

**Day 0**

1. Prepare Formation Medium (see Table 1. Preparation of Media) and warm to room temperature.
2. Prepare Seeding Medium as follows: Add 30 μL of 5 mM Y-27632 to 15 mL of Formation Medium (10 μM final concentration).
3. Use a microscope to visually identify regions of differentiation in the hPSC culture. Remove regions of differentiation by scraping with a pipette tip or by aspiration.
4. Aspirate medium from hPSC culture and wash the well with 1 mL of sterile D-PBS (Without Ca++ and Mg++).
5. Aspirate D-PBS and add 1 mL of Gentle Cell Dissociation Reagent.
6. Incubate at 37°C for 8–10 min.
  NOTE: Incubation time may vary when using different cell lines or other non-enzymatic cell dissociation reagents.
7. Using a 1 mL pipettor, gently resuspend the cells by pipetting up and down slowly 3–5 times. Transfer the cell suspension to a sterile 50 mL conical tube.
8. Rinse the well with an additional 1 mL of Seeding Medium and add the rinse to the tube containing the cells.
9. Centrifuge cells at 300 × *g* for 5 min.
10. Remove and discard supernatant. Add 1–2 mL of Seeding Medium to resuspend cells.
11. Count cells using Trypan Blue and a Hausser Scientific™ Bright-Line Hemocytometer.
12. Calculate the volume of cells required to obtain 90,000 cells/mL; add this volume of cells to an appropriate volume of Seeding Medium.
13. Add 100 μL of cell suspension from step 12 into each well of a 96-well round-bottom ultra-low attachment plate (9,000 cells/well).
  NOTE: To improve efficiency and reproducibility of organoid formation, a multi-channel pipettor is recommended for this step. Additionally, we have also found that spinning the plate at 100 x *g* for 3 min, immediately after addition of the cell suspension, can improve settling of the cells and homogeneity of organoid formation.
14. Incubate 96-well plate at 37°C. Do not disturb the plate for at least 24 h.
15. Observe plate under microscope. Small aggregates (100–200 μm) will be observed with a layer of unincorporated cells around the central aggregate.

**Day 2–5**

16. On day 2 and day 4, gently add 100 μL of Formation Medium per well. A multi-channel pipettor is recommended for this step. Incubate at 37°C.
17. On day 5, observe aggregates under a microscope. Aggregates should reach a diameter of > 300 μm (typically 400–600 μm) and exhibit round and smooth edges (see Figure 3A).
18. Proceed to section 3.1.3 (Induction).

#### 3.1.4 Induction (Day 5–7)

NOTE: Warm cultureware, medium, and reagents to room temperature (15–25°C) before use.

NOTE: As an alternative to ultra-low attachment plates, tissue culture-treated cultureware pre-treated with Anti-Adherence Rinsing Solution (STEMCELL Technologies, Canada, Catalog #07010) may be used to prevent cell attachment.

**Day 5**

1. Prepare Induction Medium (see Table 1. Preparation of Media) and warm to room temperature.
2. Add 0.5 mL of Induction Medium to each well of a 24-well ultra-low attachment plate and set aside.
3. Add 1–2 organoids to each well of the 24-well plate as follows:
  a. Using a wide-bore 200 μL pipette tip, draw up 50 μL from one well of the 96-well plate from section A to obtain organoids.
  b. Slowly and carefully eject most of the medium in the pipette tip back into the original well while retaining the organoids.
  c. Dispense the organoids into one well of the 24-well plate containing Induction Medium (prepared in step 2).
4. Place the plate in a 37°C incubator. Move the plate in several quick, short, back-and-forth and side-to-side motions 3–4 times to evenly distribute the organoids throughout the wells.
5. Incubate at 37°C for 48 h. Organoids will maintain smooth edges and develop optically translucent edges.
6. Proceed to section 3.1.3 (Expansion).

#### 3.1.5 Expansion (day 7–10)

NOTE: Matrigel^®^ embedding is necessary for proper ChP organoid formation. The addition of liquid Matrigel^®^ (Chew et al., 2022) or other non-Matrigel^®^ alternatives does not support ChP organoid formation.

**Day 7**

1. Observe organoids under a microscope.
2. Thaw Matrigel^®^ on ice at 2–8°C for 1–2 h.
  NOTE: Thaw a sufficient volume of Matrigel^®^ for 15 μL/organoid (e.g., 15 μL x 96 organoids = 1.44 mL of Matrigel^®^).
  NOTE: Keep Matrigel^®^ on ice to prevent premature polymerization. All plasticware that comes in contact with Matrigel^®^ can be chilled at −20°C for at least 30 min prior to use.
3. Prepare Expansion Medium (see Preparation of Media) and warm to room temperature (15–25°C).
4. Place the embedding surface (e.g., Organoid Embedding Sheet or Parafilm^®^) into an empty, sterile, 100 mm dish.
5. Using a wide-bore 200 μL pipette tip, draw up 25–50 μL of medium + organoids from one well of the 24-well plate and transfer to the embedding surface. Repeat this step until 12–18 organoids are collected on the embedding surface.
  NOTE: Embed no more than 12–18 organoids at a time; this will prevent the organoids from drying out and the Matrigel^®^ from prematurely polymerizing.
6. Remove excess medium from each organoid by carefully drawing up medium with a standard 200 μL pipette tip. Position the opening of the tip so that it is pointing away from the organoids to avoid drawing it up.
7. Using a pipettor with a cold 200 μL standard pipette tip, add 15 μL of Matrigel^®^ dropwise onto each organoid.
8. Using a new cold 200 μL pipette tip, reposition the organoid to the center of the droplet.
9. Incubate the 100 mm dish at 37°C for 30 min to polymerize the Matrigel^®^.
10. Use sterile forceps to grasp the embedding surface containing Matrigel^®^ droplets.
11. Position the sheet directly above one well of a 6-well ultra-low adherent plate. Using a 1 mL pipettor, draw up Expansion Medium and gently wash Matrigel^®^ droplets off the sheet and into the well. Use 3 mL of Expansion Medium/well. Repeat until all 12–16 Matrigel^®^ droplets are in the well.
12. Incubate at 37°C for 3 days. Embedded organoids will develop expanded neuroepithelia, as evidenced by budding of the organoid surface (Figure 3A).
13. Proceed to section 3.1.4 (Choroid Plexus Differentiation).

#### 3.1.6 Choroid plexus differentiation (Day 10–15)

**Day 10**

1. Prepare Choroid Plexus Differentiation Medium (see Preparation of Media) and warm to room temperature (15–25°C).
2. Using a 5 mL or 10 mL serological pipette at the slowest setting, carefully remove all medium from wells containing organoids. Do not disturb Matrigel^®^-embedded organoids.
3. Replace medium with 3 mL/well of Choroid Plexus Differentiation Medium.
4. Place the plate of organoids on an orbital shaker in a 37°C incubator.

**Day 13**

1. Perform a full-medium change with fresh Choroid Plexus Differentiation Medium.
2. Using a 5 mL or 10 mL serological pipette at the slowest setting, carefully remove all medium from wells containing organoids. Do not disturb Matrigel^®^-embedded organoids.
3. Replace medium with 3 mL/well of Choroid Plexus Differentiation Medium.
4. Return the plate to the orbital shaker in a 37°C incubator and incubate for 2 days.
5. Proceed to section 3.1.5 (Organoid Maturation).

#### 3.1.7 Choroid plexus maturation (Day 15–30+)

**Day 15**

1. Prepare Maturation Medium (see Preparation of Media) and warm to room temperature (15–25°C).
2. Using a 5 mL or 10 mL serological pipette at the slowest setting, carefully remove all medium from wells containing organoids. Do not disturb Matrigel^®^-embedded organoids.
3. Replace medium with 3 mL/well of Maturation Medium.
4. Place the plate of organoids on an orbital shaker in a 37°C incubator.
5. Perform a full-medium change every 3–4 days as follows:
  a. Tilt the cultureware.
  b. Using a 5 mL serological pipette at the slowest setting, slowly remove medium.
  c. Add 3 mL/well of fresh Maturation Medium.
  d. Return plate to the orbital shaker in a 37°C incubator.

**Day 30 +**

NOTE: By day 30, >70% of organoids should display cystic structures and can be used for downstream applications and analysis. Organoids will tend to display multiple cysts per organoid with heterogeneous morphology, which does not impact performance. Organoids with cystic structures can be grown beyond day 30, whereby the cyst may continue to balloon and enlarge. We recommend using ChP organoids between day 30–100.

6. Continue to perform full-medium changes every 3–4 days using Maturation Medium with 1% Matrigel^®^ (v/v).
  NOTE: Medium containing Matrigel^®^ must be kept at 4°C at all times to avoid the formation of precipitation.
7. ChP organoids can be assayed by cryosectioning/immunolabeling and/or by RT-qPCR (Figure 5). The following markers can be used for immunolabeling:
  • ChP: TTR, CLIC6
  • CSF: CLU, IGF2
  • Cortical plate/pre-plate/neuron: PAX6, MAP2
  NOTE: For applications requiring the transferring of cystic organoids, use a 1 mL pipette tip cut to a bore size of ∼3–5 mm. Transfer the organoids gently, as cysts may be prone to shearing if not handled carefully.
8. Proceed to section 3.2 for methods to extract CSF-like fluid from ChP organoids.

### 3.2 Method to extract CSF-like fluid from choroid plexus organoids

**FIGURE 2:**
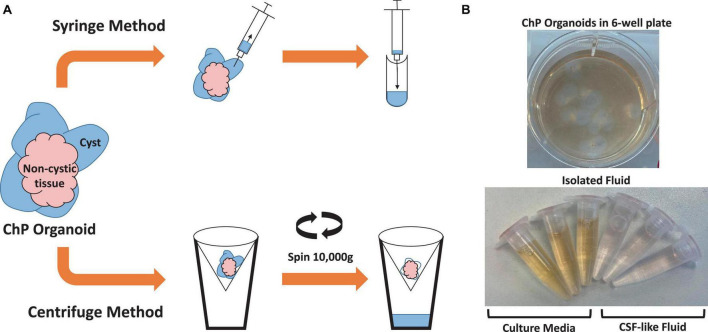
Overview of Methods for Extracting CSF-like fluid from Choroid Plexus Organoids. **(A)** Diagram of syringe method and centrifugation method to extract CSF-like fluid from ChP organoids. **(B)** Representative image of cultured day 35 ChP organoids in a 6-well plate (top), and CSF-like fluid extracted from the cysts of day 35 ChP organoids (bottom). The fluid within the cysts is distinct in color from the surrounding cell culture medium.

Two methods were used to extract CSF-like fluid from the cysts of ChP organoids (Figure 2 and Table 2). The syringe method is used for ChP organoids with cysts ≥ 1 mm in diameter, while the centrifugation method is optimal for ChP organoids with cysts of ≤1 mm in diameter.

**TABLE 2:** Comparison of CSF-like fluid extraction methods.

Extraction method	Optimal cyst size	Expected volume of CSF-like fluid
Syringe Method	≥ 1 mm diameter (medium to large)	100–1,000 μL
Centrifugation Method	≤ 1 mm diameter (small)	10–500 μL

#### 3.2.1 Syringe method

NOTE: This method is ideal for ChP organoids with cysts that are ≥ 1 mm in diameter (Figure 4B; medium to large ChP organoids)

1. Transfer the ChP organoids using a cut P1000 pipette tip (or sterile spatula) to an appropriate vessel to wash (see Figure 4B, for small to medium ChP organoids these can be transferred to a 12-well plate, for large ChP organoids these can be transferred to a 6-well plate).
2. Rinse ChP organoid using 0.5 to 1 mL of D-PBS three times to remove excess media. Note: perform wash steps gently to prevent popping of the ChP organoid cyst.
3. Use a P200 or P1000 pipette to remove as much D-PBS as possible to prevent the dilution of the CSF-like fluid.
4. Insert a 28G or smaller needle attached to a 1–3 mL syringe into a cyst and slowly extract CSF-like fluid.
  NOTE: If possible, aim for the middle of the cysts. Holding the tube at eye-level, within the biosafety cabinet, can facilitate this.
5. Expel the CSF-like fluid into a new microcentrifuge tube and store at −20°C to −80°C for subsequent analysis.
  NOTE: The total volume of CSF-like fluid extracted from each organoid can range from 10 to 1,000 μL, depending on the method.

#### 3.2.2 Centrifugation method

NOTE: This method is ideal for ChP organoids with a cyst diameter less than 1 mm or when multiple ChP organoids need to be processed per sample (Figure 4B; small to medium ChP organoids). It’s not recommended to use this method with organoid cyst diameter greater than 1 mm due to dimensions of the Amicon Ultra-0.5 mL Centrifugal Filter. The Amicon Ultra-15 Centrifugal Filter Unit (Catalog #UFC910008) may allow processing for larger ChP organoid dimensions but we have not tested this unit for this specific application.

1. Transfer the ChP organoids using a cut p1000 pipette tip (or sterile spatula) to an appropriate vessel to wash.
  NOTE: Small to medium ChP organoids can be transferred to a 12-well plate, and large ChP organoids can be transferred to a 6-well plate (see Figure 4B).
2. Rinse the ChP organoid using 0.5 to 1 mL of D-PBS twice to remove excess medium.
3. Transfer ChP organoid(s) to a Amicon Ultra-0.5 mL Centrifugal Filter (100 kDa cut-off). Remove as much excess D-PBS as possible to prevent dilution of the CSF-like fluid.
  NOTE: We recommend loading no more than 2–3 organoids per tube to avoid clogging the filter.
4. Centrifuge tube at 10,000 x *g* for 2 min.
5. Collect the CSF-like fluid in the flow-through and store at −20°C to −80°C for subsequent analysis.

Deflated ChP organoid tissue from either extraction method can be used as a control in subsequent experiments and can be stored in 4X Laemmli Sample Buffer for Western Blot or lysed using Buffer RLT (Qiagen Catalog #79216) for RNA extraction.

### 3.3 Western blot analysis of CSF-like fluid

We used Western blot analysis to identify proteins known to be expressed in CSF, such as clusterin and IGF-2. Conditions and antibodies used will need to be optimized for other applications.

#### 3.3.1 Buffer preparation

1. Prepare 1X Running Buffer by diluting 10X Tris/Glycine/SDS Running Buffer to a 1X concentration in dH_2_O. Mix thoroughly.
2. Prepare Transfer Buffer with 48 mM Tris, 39 mM glycine, 0.0375% SDS, and 20% methanol as follows:
  a. Add 5.8 g of Tris–HCl, 2.9 g of glycine, 3.75 mL of 10% SDS, and 200 mL of methanol to 800 mL of D-PBS.
  b. Mix thoroughly and store at 2–4°C.
3. Prepare Blocking Buffer with 5% BSA and 0.2% TWEEN^®^ 20 as follows:
  a. Add 0.5 g of BSA and 20 μL of TWEEN^®^ 20 to 10 mL of D-PBS.
  b. Mix the solution thoroughly after adding each component.
4. Prepare Wash Buffer by adding 1 mL of TWEEN^®^ 20 to 1 L of D-PBS (0.1% final concentration).

#### 3.3.2 Sample preparation

1. Prepare 4X reducing Laemmli Sample Buffer by adding 100 μL of 2-mercaptoethanol to 900 μL of 4X Laemmli Sample Buffer.
2. Add 1 part 4X Laemmli Sample Buffer to 3 parts sample volume (e.g., ∼20 μL of CSF-like fluid or spent medium control + ∼7 μL of 4X Laemmli Sample Buffer).
  NOTE: We have found that the protein concentration of CSF-like fluid ranges from 0.2 to 0.5 mg/mL; adjust the dilution according to your application.
3. Heat samples at 95°C for 5 min.

#### 3.3.3 SDS-PAGE

1. Prepare four 15% Mini-PROTEAN^®^ TGX™ Precast Gels and electrophoresis apparatus in Running Buffer.
2. Load protein standards and samples onto gels.
3. Run SDS-PAGE at 200 V constant voltage for 60 min.
  NOTE: Observe molecular weight markers and sample dye front to ensure the sample does not run off the bottom of the gel during electrophoresis.
4. Rinse gels twice with distilled water.
5. Equilibrate the gels in cold (2–8°C) Transfer Buffer for 15 min.

#### 3.3.4 Transfer

1. Submerge the 0.2 μm PVDF membrane in methanol for 2 min. Remove from the methanol and incubate in cold Transfer Buffer (equilibrated to 2–8°C) for at least 5 min.
2. Soak 2 pieces of extra-thick blotting paper in Transfer Buffer.
3. Assemble transfer sandwich (gel, PVDF membrane, and blotting papers) as per instrument instruction.
4. Run the semi-dry transfer at 10 V constant voltage for 90 min.

#### 3.3.5 Blotting

1. Remove the PVDF membrane from the transfer apparatus and rinse twice with D-PBS.
2. Incubate PVDF membrane in Blocking Buffer at room temperature (15–25°C) for 1 h with agitation.
3. Wash the membrane 2X for 5 min with Wash Buffer.
4. Dilute the primary antibodies in Blocking Buffer as follows:
  i. 1:250 Anti-IGF2 Antibody (unconjugated, rabbit polyclonal; Abcam Catalog #ab9574)
  ii. 1:250 HRP Anti-Clusterin Antibody (HRP-conjugated, mouse monoclonal; BioLegend Catalog #848703)
5. Incubate the membrane with primary antibodies at room temperature for 30 min with agitation, followed by 4°C overnight without agitation.
6. Wash membrane 4X for 5 min with Wash Buffer.
7. Dilute secondary antibody in Blocking Buffer as follows:
  i. For IGF-2 blot: 1:2000 Goat Anti-Rabbit IgG-HRP (Abcam Catalog #ab205718).
  ii. For clusterin blot: No secondary antibody needed since primary clusterin antibody is HRP-conjugated.
8. Incubate the membrane for 1 h at room temperature with agitation.
9. Wash membrane 4X for 5 min with Wash Buffer.
10. Prepare enhanced chemiluminescence (ECL) HRP substrate solution using Clarity Western ECL Substrate as follows:
  a. Mix Clarity Western Peroxide Reagent and Clarity Western Luminol/Enhancer Reagent at a 1:1 ratio.
  b. Mix thoroughly.
11. Immerse the membrane in the substrate solution for 1 min at room temperature. Remove membrane and blot away excess liquid.
12. Image blots and export for analysis.

### 3.4 Proteomic analysis by mass spectrometry

#### 3.4.1 Sample preparation

1. Use Nanodrop to measure protein concentration. CSF fluid has a protein concentration ranging from 0.2 to 0.5 mg/mL, and cell culture medium is typically > 1 mg/mL.
2. Use 4 μg of CSF samples or 10 μg of medium samples for in-solution digestion. Samples are diluted with 50 mM ammonium bicarbonate (pH 8) to adjust pH.
3. Add 0.02 μg of dithiothreitol (DTT) per μg of sample protein. Incubate samples at room temperature (15–25°C) temperature for 30 min.
4. To alkalize samples, add 0.1 μg of chloroacetamide (CAA) per μg of sample protein. Incubate samples at room temperature for 20 min in the dark.
5. Digest samples by adding 0.02 μg of trypsin per μg of protein sample and incubate at 37°C overnight.
6. The next day, acidify samples with 10% TFA to stop digestion (final pH < 2.5).
7. Desalt samples with C18 columns and stage-tipped with either 4 mm C18 or 10 mm C18. Stage tips are conditioned with 100% methanol, equilibrated with 0.2% TFA, and loaded with samples, followed by washing twice with 200 μL of 0.2% TFA.
8. Samples are eluted twice with 80 μL of 40% acetonitrile, 0.1% formic acid and subsequently dried by vacuum.

#### 3.4.2 Liquid chromatography-mass spectrometry (LC-MS) analysis

1. Reconstitute dried samples in 0.5% acetonitrile and 0.1% formic acid and measure protein concentration on a NanoDrop™ One spectrophotometer (Thermo Scientific) using the A205 Scope method (205 nm absorbance, 340 nm baseline correction).
2. Inject 25 ng of peptides from CSF samples or 80 ng of peptides from culture media samples onto a nanoElute^®^ Nano-Flow UHPLC system (Bruker Daltonics) with a Gen2 25 cm Aurora Series analytical column with CaptiveSpray Insert (CSI; Ion Opticks #AUR2-25075C18A-CSI).
3. Heat the analytical column to 50°C using a Bruker Column Toaster.
4. Set LC-MS conditions as follows:
  a. Buffer A consists of 0.1% aqueous formic acid and 0.5% acetonitrile in water.
  b. Buffer B consists of 0.1% aqueous formic acid and 0.5% water in acetonitrile.
5. Before each run, condition analytical column with 4 column volumes of Buffer A. A standard 30-min gradient was performed as follows:
  a. *t* = 0 min, 2% B; *t* = 15 min, 12% B; *t* = 30 min, 33% B; *t* = 30.5 min, 95% B.
  b. Hold at 95% B from *t* = 30.5 min to *t* = 38.22 min.
6. Analyze peptides by time-of-flight (TOF) trapped ion mobility spectrometry (TIMS) using a timsTOF Pro2 mass spectrometer (Bruker Optics, Ltd.).
  a. Perform analysis at a flow rate of 0.30 μL/min. The NanoElute^®^ thermostat temperature was set to 7°C. Operate Captive Spray ionization source at 1,800 V capillary voltage, 3 L/min drying gas, and 180°C drying temperature.
7. During analysis, operate timsTOF Pro 2 with parallel accumulation-serial fragmentation (PASEF) scan mode for data-dependent acquisition (DDA).
8. Collect MS and tandem MS (MS/MS) results in positive mode, separated by mass-to-charge ratios from m/z = 100–1,700 Th, and an ion mobility range (1/k0) from 0.7 to 1.35 V*s/cm^2^.
9. Apply polygon filter to the mass-to-charge and the ion mobility plane to include the most likely peptide precursors and to reduce singly-charged background ions.
10. The TIMS-MS scan was set at 100 ms ramp time and accumulation time at a rate of 9.42 Hz (100% duty cycle). Parent ions were then excluded from MS/MS for the next 0.4 min and reconsidered if their intensity increased more than 4 times.
11. For each TIMS cycle, 5 PASEF MS/MS scans were recorded (total cycle time of 0.64 s).
  a. The isolation windows for MS/MS were set at 2.07 m/z at < 400 m/z and 3.46 m/z at > 1,000 m/z. The collision energy was ramped linearly as a function of mobility value from 27 eV at 1/k0 = 0.7 V⋅s/cm^2^ to 55 eV at 1/k0 = 1.35 V⋅s/cm^2^. The timsTOF Pro 2 was run with timsControl (client version 3.0.0; Bruker Daltonics). LC and MS were controlled with Compass HyStar 6.0 software (6.0.30.0; Bruker). The mass-to-charge ratio and ion mobility value were calibrated based on three selected ions from Agilent ESI-Low Tuning Mix ions [m/z (Th), 1/k0 (Th): 622.0290, 0.9915; 922.0098, 1.1986; 1221.9906, 1.3934]. The mass accuracy was typically within 4 ppm and is not allowed to exceed 7 ppm.

#### 3.4.3 Data analysis

Raw data was searched on Byonic (v4.0.12) against a combined fasta of human and bovine, downloaded from UniProt (human fasta was released 2021/01 with 20391 entries; bovine fasta was released 2017/07 with 23973 entries). Precursor and fragment mass tolerance were set at 50 ppm mass error. Carbamidomethyl at C was set as fixed modification; deamidation at Q and oxidation at M were set as variable modification.

## 4 Results

### 4.1 Choroid plexus organoids

**FIGURE 3:**
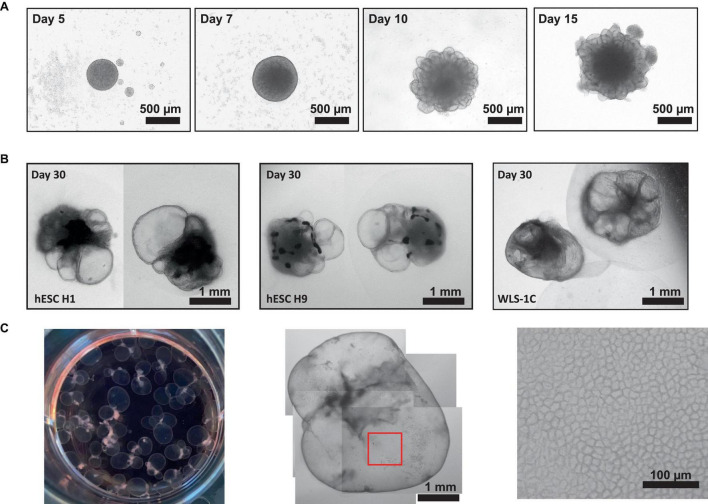
Representative Morphology at Different Stages of ChP Organoid Generation. **(A)** Representative morphology of ChP organoids after organoid formation (day 5), induction (day 7), expansion (day 10) and ChP differentiation (day 15). **(B)** Morphology of cystic day 30 H1-, H9-, and 1C-derived ChP organoids. **(C)** Left: an image of a 6-well plate of day 50 ChP organoids. Middle: representative morphology of a day 50 ChP organoid composed primarily of a fluid-filled cyst. Right; 100X magnification of surface of the cyst (red box shown in middle image) shows tightly packed cells displaying a cuboidal morphology.

**FIGURE 4:**
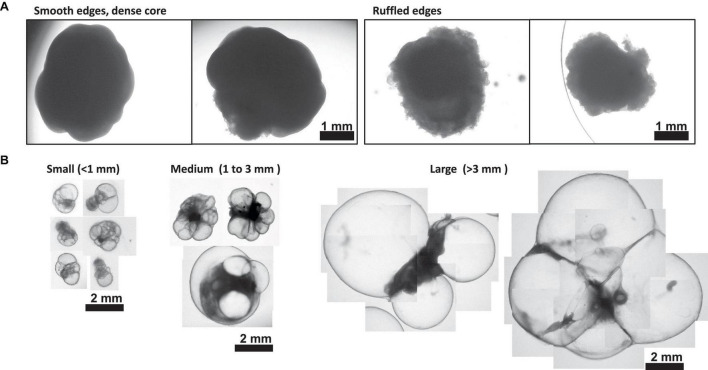
Morphology of Day 30 + Choroid Plexus Organoids. **(A)** Representative images of poor day 50 ChP organoid morphology. Left: organoids displaying smooth edges and dense core, indicative of cortical organoid tissue. Right; organoids displaying ruffled edges, indicative of a misoriented ChP organoid that does not form a cyst. **(B)** Morphology of small (<1 mm in diameter), medium (1–3 mm diameter) and large (>3 mm in diameter) cystic ChP organoids between day 40–50. Cystic ChP organoids may develop multiple cysts per organoid.

**FIGURE 5:**
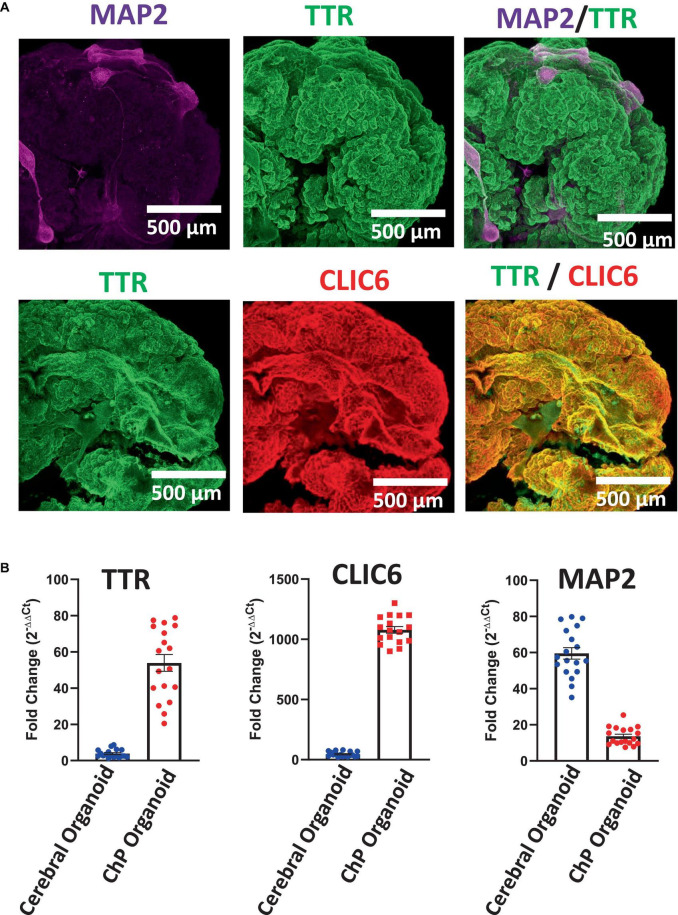
Characterization of Day 50 ChP Organoids. **(A)** Immunostaining of whole ChP organoids revealed high expression of TTR (green) and CLIC6 (red), but low overall expression of MAP2. **(B)** RT-qPCR analysis confirmed upregulation of TTR and CLIC6 in ChP organoids while MAP2 expression is low in comparison to cerebral organoids (Pham et al., 2018; Chew et al., 2022) (average ± SEM; *n* = 6 cell lines, 3 experiments per cell line). Each data point is an average of data from 3 organoids. Data was normalized to 18S/TBP and compared to an undifferentiated hPSC control. Expression of all three genes was significantly distinct between cerebral and ChP organoids (*T*-test, *p* < 0.0001).

Here, we have presented a method for generating ChP organoids derived from multiple cell lines using a commercially available kit. Figures 3A, B provide representative images from the five stages of ChP organoid generation (Figure 1). We have obtained a success rate of over 70% (88% ± 0.09 SD success in generating cyst-displaying organoids using *n* = 7 cell lines over 3 experiments per cell line). The cyst portion of the organoid is made of cuboidal ependymal cells which display tight packing (Figure 3C, right panel). Figure 4A shows morphology of non-cystic organoids which may arise in culture. Some organoids display smooth and dense centers (Figure 4A, left panel) indicating formation of cortical organoids. On the other hand, organoids with ruffled edges (Figure 4A, right panel) usually express high levels of ChP markers but exhibit misorientation in their apico-basal polarity, resulting in the absence of cyst formation. In some cases, these types of organoids can be re-embedded into a Matrigel^®^ droplet, and a cyst may form after several weeks in culture. Figure 4B highlights the diversity of cystic ChP organoid morphologies. Usually, ChP organoids display more than one cyst per organoid and can be broadly classified based on the cyst diameter; small (<1 mm in diameter), medium (1–3 mm diameter) or large (>3 mm in diameter) cysts. The corresponding cyst diameter can be used to choose the most optimal CSF-like fluid extraction method (section 3.2). Figure 5 shows characterization of ChP organoids using whole-organoid immunostaining and quantitative reverse transcription polymerase chain reaction (RT-qPCR). ChP organoids showed upregulation of ChP ependymal cell markers transthyretin (TTR) and chloride intracellular channel 6 (CLIC6) but do not express the neuronal marker microtubule-associated protein (MAP2).

### 4.2 Analyzing CSF-like fluids

**FIGURE 6:**
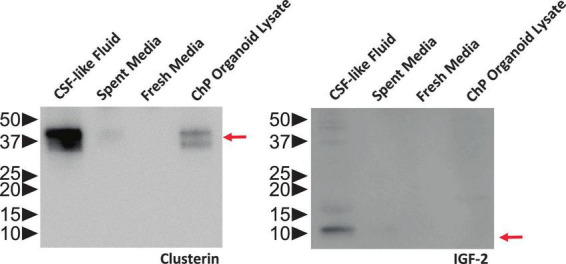
ChP Organoids Secret CSF-Like Fluid. Representative Western blot showing CSF-like fluid extracted from R038 iPSC-derived ChP organoids contained clusterin (left) and IGF-2 (right), two proteins found in abundance in human CSF. Molecular weight ladder indicating protein size in kDa is labeled on the left. Red arrows indicate the expected size of target protein on the blot.

**FIGURE 7:**
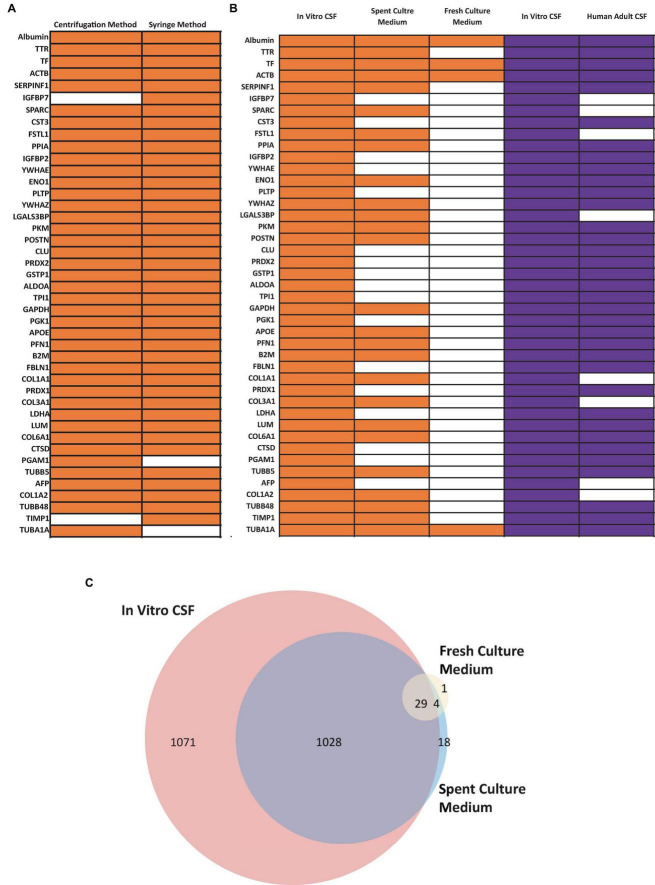
Confirmation of CSF Proteins in CSF-like Fluid by Mass Spectrometry (MS). **(A)** Comparison human CSF proteins identified in the extracted CSF-like fluid from the syringe or centrifugation method (day 30 organoid, *n* = 1 extraction per condition) **(B)** Comparison of human CSF proteins from CSF-like fluid, spent culture medium, and fresh culture medium compared to published dataset (purple) from Pellegrini et al. (2020). Three in-house samples were collected from day 30–50 ChP organoids. Orange bars indicate that the corresponding protein was detected in at least two out of three samples, while the white bars indicate that the protein was detected in ≤ 1 sample. **(C)** Venn diagram comparing total proteins detected in *in vitro* CSF (red), spent culture medium (blue), and fresh culture medium (yellow). A total of 1,028 proteins detected in *in vivo* CSF were also detected in spent culture medium (purple).

The fluid from cystic ChP organoids can be extracted using either the syringe or centrifugation methods described (section 3.2). Extracted CSF-like fluid was found to be colorless compared to cell culture medium (Figure 2). Protein concentration of CSF-like fluid was measured to be between 0.2–0.5 mg/mL, whereas cell culture medium is > 1 mg/mL. CSF-like fluid can be analyzed using Western blot (Figure 6), in this case we observed clusterin and IGF-2, two proteins found in primary CSF (Watanabe et al., 2012). CSF-like fluid was also analyzed using mass spectrometry for protein identification. Here we identified a broad subset of proteins that are also found in high abundance in primary CSF (Figures 7A, B). In comparing the centrifugation method versus the syringe method, we obtain a high degree of overlap in proteins between these two methods (Figure 7A). Importantly, when we compare the CSF-like fluid obtained from our method to the published datasets (Pellegrini et al., 2020) we identified the same subset of proteins which also closely overlaps with human adult CSF. In comparison to cell culture medium, >1,000 proteins did not overlap, indicating a compartmentalization of this subset of proteins into the cyst of the organoid (Figure 7C).

## 5 Discussion

Choroid plexus organoids have emerged as a promising tool in the field of neuroscience and neurobiology, offering researchers the opportunity to study the ChP—a specialized structure responsible for the production of cerebrospinal fluid—in a more accurate and controlled manner. Here we describe a method to generate hPSC-derived ChP organoids using a commercially available kit. Our method generates ChP organoids which form cysts and express appropriate markers of primary ChP; TTR and CLIC6. The fluid from ChP organoids can be extracted and analyzed for protein composition. We have shown that the CSF-like fluid contains proteins native to primary CSF. Using a sensitive mass spectrometry approach we have been able to detect thousands of proteins unique to primary CSF. Interestingly, about half of these proteins were identified only in the CSF-like fluid, and not in cell culture medium, indicating a compartmentalization of these proteins. This observation is indicative of a barrier formed by the ChP-specific ependymal cells lining the cyst of the organoid. Indeed, previous work has shown that ChP organoids express selective transporters and tight junction proteins which contributed to a selective barrier which regulated small molecule efflux. (Pellegrini et al., 2020) taken together, ChP organoids are a novel system to obtain CSF-like fluid and to study the ChP barrier.

While ChP organoids provide valuable insights into the development and function of the ChP, it is essential to acknowledge their limitations. ChP organoids are derived from PSCs, which are capable of differentiating into multiple cell types. However, these organoids lack cell types that are not derived from the ectoderm lineage, including endothelial or immune cells. The ChP functions as an important barrier and does so in combination with endothelial cells to form the blood-CSF barrier. ChP organoids, in their current form, lack a functional vasculature network to fully recapitulate this specific barrier, despite demonstrating its utility to predict drug permeability (Pellegrini et al., 2020). Exploring methods to integrate endothelial cells into ChP organoids will be an important next step in improving this model. Indeed, vascularizing neural organoids, either *in vivo* (Mansour et al., 2018; Revah et al., 2022) or through co-culture with exogenous endothelial cells (Pham et al., 2018; Cakir et al., 2019), has shown to improve functionality. For the immune cell component, macrophages and microglia are found to interact with and regulate neuroinflammation and pathogen surveillance within the ChP (Saunders et al., 2023). This highlights an opportunity to co-culture ChP organoids with these missing cell types to better recapitulate these cell-cell interactions.

Transcriptionally, ChP organoids have been found to represent fetal ChP (Pellegrini et al., 2020). Although useful for modeling features of the early developing ChP, ChP organoids may not be as useful to model post-natal phenotypes or mechanisms. Indeed, the CSF-like fluid produced by ChP organoids appears to be more fetal in composition, expressing fetal markers such as IGF-2 which are not found in adult CSF (Pellegrini et al., 2020). Despite these limitations, ChP organoids offer unique opportunities for investigating ChP development, ability to obtain CSF fluid for biomarker discovery and as a screening tool to test crossing of candidate drugs along a biologically relevant barrier. Future improvements to this system will likely include incorporating additional components such as vascularization to enhance their relevance to the native ChP or improving the maturation state of this tissue model. Overcoming these limitations will contribute to the further advancement and application of ChP organoids in neuroscience research, potentially providing novel insights into brain disorders and therapeutic interventions.

## Data availability statement

The original contributions presented in this study are included in this article/supplementary material, further inquiries can be directed to the corresponding author.

## Ethics statement

Ethical approval was not required for the studies on humans in accordance with the local legislation and institutional requirements because only commercially available established cell lines were used.

## Author contributions

LC and EK conceived and designed the analysis. LC, EM, and JR collected the data or analysis tools. LC and EM performed the analysis. LC, EM, JR, SP, and EK wrote the manuscript. All authors contributed to the article and approved the submitted version.
